# Somatostatin Receptor Targeted PET-CT and Its Role in the Management and Theranostics of Gastroenteropancreatic Neuroendocrine Neoplasms

**DOI:** 10.3390/diagnostics13132154

**Published:** 2023-06-24

**Authors:** Aadil Adnan, Sandip Basu

**Affiliations:** 1 Radiation Medicine Centre, Bhabha Atomic Research Centre, Tata Memorial Centre Annexe, JerbaiWadia Road, Parel, Mumbai 400012, India; doc.adnanaadil@gmail.com; 2 Homi Bhabha National Institute, Mumbai 400094, India

**Keywords:** SSTR PET-CT, somatostatin receptor, neuroendocrine neoplasm, theranostics, gastroenteropancreatic, precision oncology, ^68^Ga-DOTA-TATE PET-CT

## Abstract

Somatostatin receptor (SSTR) agonist-based Positron Emission Tomography-Computed Tomography (PET-CT) imaging is nowadays the mainstay for the assessment and diagnostic imaging of neuroendocrine neoplasms (NEN), especially in well-differentiated neuroendocrine tumors (NET) (World Health Organization (WHO) grade I and II). Major clinical indications for SSTR imaging are primary staging and metastatic workup, especially (a) before surgery, (b) detection of unknown primary in metastatic NET, (c) patient selection for theranostics and appropriate therapy, especially peptide receptor radionuclide therapy (PRRT), while less major indications include treatment response evaluation on and disease prognostication. Dual tracer PET-CT imaging using SSTR targeted PET tracers, viz. [^68^Ga]Ga-DOTA-Tyr3-Octreotate (DOTA-TATE) and [^68^Ga]Ga-DOTA-NaI3-Octreotide (DOTA-NOC), and fluorodeoxyglucose (FDG), have recently gained widespread acceptance for better assessment of whole-body tumor biology compared to single-site histopathology, in terms of being non-invasive and the ability to assess inter- and intra-tumoral heterogeneity on a global scale. FDG uptake has been identified as independent adverse risk factor in various studies. Recently, somatostatin receptor antagonists have been shown to be more sensitive and specific in detecting the disease. The aim of this review article is to summarize the clinical importance of SSTR-based imaging in the clinical management of neuroendocrine and related tumors.

## 1. Introduction

Neuroendocrine neoplasms (NENs) are a heterogeneous group of malignant tumors derived from neural and endocrine cells; they can involve many different parts of the body, but most commonly are located in gastrointestinal tract. Gastro-entero-pancreatic neuroendocrine neoplasms (GEP-NEN) are heterogeneous group of malignant tumors, predominantly located in the intestine (small intestine more frequent that the large intestine) and pancreas, with liver being the most common metastatic site [1,2]. Most of the NENs are characterized by slow indolent growth leading to late diagnosis, high prevalence and approximately half of the cases are metastatic at diagnosis [3,4,5,6].

These are classified depending on (a) histopathological grade and proliferative (mitotic) activity, by the World Health Organization (WHO) into (A) well-differentiated grade I, II, and III neuroendocrine tumour (NET) with proliferative index and mitotic activity (< 3%, <2%; 3 to 20%, 2 to 20% and >20%, respectively) and (B) poorly-differentiated neuroendocrine carcinoma (NEC) with mitotic and Ki-67 indices >20% and poorly differentiated histology. The 2019 WHO classification of NET has also divided NENs as NET and NEC based on genetic mutations: mutations in *MEN1*, *DAXX*, and *ATRX* are entity-defining for well-differentiated NETs, whereas NECs usually have *TP53* or *RB1* mutations [7,8]; (b) functioning status depending upon secretion or non-secretion of peptide hormones as functioning and non-functioning NENs; and (c) embryological origin as foregut, mid-gut, and hind-gut derivatives. 

The diagnosis of NET is characterized by detection of immunohistochemical markers—synaptophysin, chromogranin A, and neuron-specific enolase. Over the years, the role of nuclear medicine has grown to play a central role in the diagnosis of NEN after the identification of somatostatin (SST) in 1973 and 5 types of somatostatin receptors (SSTRs 1 to 5) in the early 1990s. The discovery of somatostatin receptors has opened up new avenues for diagnosis, staging, and treatment of NENs, especially the well-differentiated type. Somatostatin-receptor-based imaging using synthetic somatostatin agonists (SSA) was started in 1994 with [^111^In] In-pentetreotide (Octreoscan) being the first Food and Drug Association (FDA) approved and commercially marketed radiopharmaceutical [9,10,11,12]. Although there was wide acceptance of Octreoscan for NEN imaging, the radiopharmaceutical had many limitations—less favorable tumor-to-background ratio, moderate affinity for receptors, and high gamma energy causing more background noise and high radiation absorbed dose to the patient. To a great extent, these limitations have been alleviated with the advent of the next generation of SSA labelled with positron emitter radio-metal [^68^Ga] to be used with PET-CT [13,14]. Recent advancements in the detection and mapping of SSTR expression in vivo has opened avenues for targeting the same for therapeutic benefits and personalized management. After securing FDA approval in January 2018, peptide receptor radionuclide therapy (PRRT) using Lutetium-177 DOTA-TATE ([^177^Lu]Lu-DOTA-TATE) has gained widespread acceptance as one of the frontline treatments in metastatic/inoperable neuroendocrine tumors (NET).

In this review, we have endeavored to summarize, in brief, the utility of somatostatin-receptor-based molecular targeted imaging and how this imaging modality is stationed in the present-day clinical scenario for patient management and theranostic applications, along with recent trends and advancements in imaging of neuroendocrine tumors that are currently on the horizon.

## 2. Radiopharmaceuticals for SSTR-PET Imaging

### 2.1. Somatostatin Receptor Agonists

Somatostatin receptors are G-protein coupled receptors (GPCR) binding to somatostatin neuropeptides, a paracrine secreted by gastro-intestinal and brain cells. Presently, various types of somatostatin agonists and few antagonists are available for clinical and/or experimental use. The common radio-pharmaceuticals for clinical use are–[^68^Ga]Ga-DOTA-Tyr3-Octreotate (DOTA-TATE), [^68^Ga]Ga-DOTA-Phe1-Tyr3-Octreotide (DOTA-TOC), and [^68^Ga]Ga-DOTA-NaI3-Octreotide (DOTA-NOC). These three radiopharmaceuticals differ slightly in their pharmacokinetic properties, mainly due to different affinities for SSTR subtypes; while DOTA-TATE is SSTR 2 specific, DOTA-NOC has affinity towards SSTR 2, 3, and 5 and DOTA-TOC has affinity towards SSTR 2 > 5 [15,16,17,18]. Despite different receptor affinity, there is no clinically significant difference and there are ample data to support high accuracy of SSTR PET-CT for detecting lesions as compared to conventional imaging and somatostatin-receptor scintigraphy [19,20,21,22,23,24,25]. 

[18F]-Fluorine (^18^F) labelled radio pharmaceuticals are recently being developed with the following advantages: long half-life, no need of in-house generators or cyclotron, and relatively low positron energy which leading to better spatial resolution than [^68^Ga]. Al [18F]F-NOTA-Octreotide is one recent promising radiopharmaceutical demonstrating high affinity for SSTR2, favorable biodistribution, high tumor uptake, better spatial resolution, and is proven to be safe for clinical applications [5,26,27,28,29,30,31].

[^64^Cu]Cu-DOTATATE is a cyclotron produced positron emitter that can be manufactured on a large scale, yields similar detection rates as [^68^Ga]-based SSTR-PET agents and has better pharmacokinetic properties such as (i) longer half-life (~12.7 h), (ii) relatively low positron energy (0.65 vs. 1.9 MeV) leading to shorter positron range (mean −0.56 vs. 3.5 mm), and (iii) higher spatial resolution enabling better detection of smaller lesions. [^64^Cu] hence permits delayed serial imaging with important implications for personalized dosimetry in peptide receptor radionuclide therapy (PRRT) and radio-guided surgery using a hand-held positron probe [32,33].

### 2.2. Somatostatin Receptor Antagonists

In opposition to the general belief that agonists will be more suitable as an imaging agent since they are internalized, a recent in vitro study using SSTR-3 antagonist has demonstrated that the antagonist detected 76-fold more sites of binding as against SSTR-3 agonist [34]. Few recent studies have shown that radiolabeled SSTR antagonists produce superior images than radiolabeled SSTR agonists [34,35]. Recently, few studies with small number of NET patients demonstrated radiolabeled SSTR-2 antagonists, e.g., [^111^In]In-DOTA-BASS and [^68^Ga]Ga-OPS202 ([^68^Ga]Ga-NODAGA-JR11) have demonstrated superior images and higher sensitivity compared to radiolabeled SSTR-2 agonists [34,35,36,37,38]. This led to the opinion that [^177^Lu] labelled antagonist in PRRT may be utilized instead of [^177^Lu]-labelled agonists. [^177^Lu]Lu-DOTA-JR11 provided 1.7-to-10.6-fold higher tumor uptake as compared to agonists, resulting in partial remission in half of the enrolled patients [35].

### 2.3. Appropriate Use Criteria

Appropriateness use criteria (AUC) defines set of scenarios finalized by the representatives of international societies concerned with management of NETs. The process of determining AUC was modelled after RAND/UCLA appropriateness method including a list of common scenarios encountered in NET management, a systematic review of evidence related to these scenarios and development of an appropriateness score for each scenario using a modified Delphi process [39,40]. The workgroup identified 12 scenarios for patients with NETs and scored each scenario as “appropriate”, “may be appropriate”, or “rarely appropriate” on a scale from 1 to 9; where 7 to 9 are appropriate, 4 to 6 are considered may be appropriate, and 1 to 3 indicate that the use is rarely appropriate and is not considered acceptable.

Various scenarios included in the AUC with respective scores in decreasing order are as follows (Table 1):

(a) **Appropriate:** Initial staging after histologic diagnosis of NET-9; localization of primary tumor in patients with known metastatic NET but unknown primary-9; selection of patients for SSTR targeting PRRT-9; staging NETs before planned surgery-8; evaluation of mass suggestive of NET and not amenable to endoscopic or percutaneous biopsy-8; monitoring of NETs seen predominantly on SSTR PET-8; evaluation of patients with biochemical evidence and symptoms of NET without evidence on conventional imaging (CI) and without prior histological diagnosis of NET-7; restaging at the time of clinical or laboratory progression without progression on CI-7; and new indeterminate lesion on CI with unclear progression-7.
(b) **May be appropriate:** Restaging of patients with NETs at initial follow up after resection with curative intent-6; selection of patients with non-functional NETs for SSA treatment-6; and monitoring in patients with NETs seen on both CI and SSTR PET with active disease and no clinical evidence of progression-6.

### 2.4. Clinical Utility of SSTR-PET

**Figure 1:**
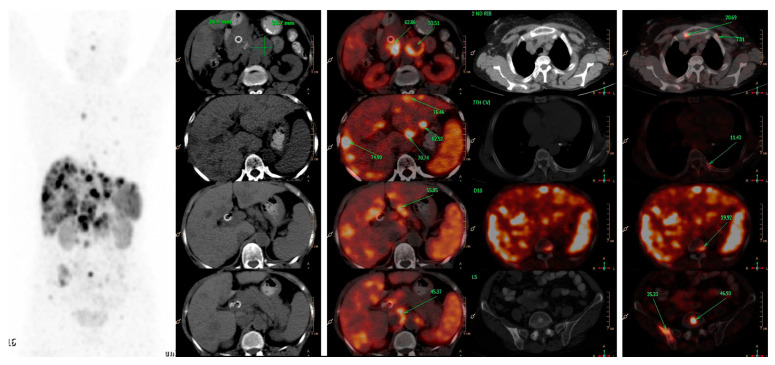
A 66-years-old female patient, presented with features of obstructive jaundice. Triphasic CECT scan abdomen showed large hypodense SOL in head of pancreas encasing CBD with significant upstream dilatation of biliary tree with multiple hypodense bilobar hepatic metastases and multiple abdominal & retroperitoneal lymph nodes. Patient was referred for PRRT and ^68^Ga-DOTATATE PET-CT was done which confirmed above-mentioned triphasic CE-CT scan findings and showed many new skeletal and marrow lesions at multiple skeletal sites. Hence is SSTR-based PET-CT represents a better modality for metastatic workup than conventional imaging.

**Detection and Initial Staging:** When compared with conventional imaging, SSTR-PET offers multiple advantages in terms of detection rate of primary and metastatic disease and change in management (observed in 44% upfront and in 9% of patients who have undergone previous somatostatin receptor scintigraphy with Octreoscan) ([41], Figure 1). Bauckneht et al., in their review and meta-analysis including 1143 patients of pancreatic NET, demonstrated a pooled sensitivity and specificity of 79.6% (95% CI—71 to 87%) and 95% (95% CI—75 to 100%), respectively; heterogeneity of 59.6% and 51.5%, and on a per patient and per lesion basis, the pooled detection rates of primary lesion were 81% (95% CI—65 to 90%) and 92% (95% CI—80 to 97%), respectively [42]. Geijer and Breimer, in their meta-analysis on 2015 patients, demonstrated pooled sensitivity of 93% (95% CI—91 to 94%) [43]. Despite theoretical advantage, conflicting results were observed in detection with contrast enhanced vs. non-enhanced PET-CT for the detection of primary and metastatic disease. While Kazmierczak et al. showed 50% and 30% improvement in sensitivity and accuracy, Mayerhoefer et al. found only moderate improvement in sensitivity and hardly any change in specificity [44,45]. Similarly, combining PET and MRI has theoretical advantage of high soft tissue contrast for MRI and metabolic data from PET.

**Figure 2:**
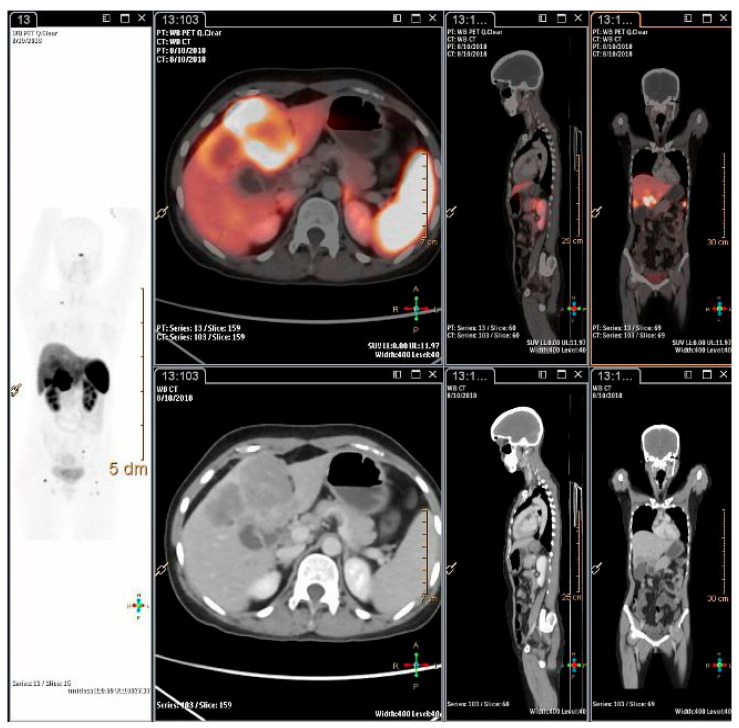
A 26-years-old lady, presented with pain abdomen and was evaluated for the same. CECT abdomen showed multiple liver lesions and abdominal lymph nodes. Biopsy and IHC from liver lesion showed high grade neuroendocrine carcinoma, large cell variant (Ki-67 index 80%). She was referred for opinion for PRRT. ^68^Ga-DOTATATE PET-CT scan showed intensely SSTR expressing polypoidal gall bladder mass infiltrating into inferior surface of liver with SSTR expressing multiple liver lesions, abdomino-pelvic lymph nodes and SSTR expressing lesion in head of right femur.

**Detection of unknown primary:** Carcinoma of unknown primary (CUP) accounts for 3 to 5% of all malignancies and is divided into following subtypes based on histological subtypes: adenocarcinoma (80 to 85%), squamous cell carcinoma (5 to 10%), and neuroendocrine tumor (2 to 4%) [46]. Neuroendocrine tumors of unknown primary (CUP-NETs) are primary tumors with undetermined origin among metastatic NETs and accounts for 11 to 22% of NETs ([47,48]; Figure 2). Ma et al., in their meta-analysis of 484 patients, demonstrated pooled sensitivity and specificity of SSTR imaging in identifying CUP-NETs as 82% and 55%, respectively [49]. The area under the receiver operating curve (ROC) was 69% and pooled detection rate for CUP-NETs was 61%. SSTR PET-CT identified most metastases in liver (57.9%) followed by lymph nodes (22.8%), bones (12.8%), lung (2.8%), and others (1.7%). Sampathirao et al., in their study on 51 CUP-NET patients using “dual tracer” PET-CT with [^68^Ga]GA-DOTATATE and [^18^F]-FDG, demonstrated sensitivity of 60.78%, whereas overall lesion detection sensitivity was 96.87% [50]. Delpassand et al., in their study, found [^64^Cu]Cu-DOTATATE to be an effective radiopharmaceutical in detecting NET lesions with sensitivity and specificity of 90.9% and 96.6%, respectively [32].

**Treatment planning and patient selection for PRRT:** Surgery is the only approach that can offer complete cure, and hence, should be a preferred option wherever applicable [51]. Surgical resection in NET should be decided considering symptoms, grade (discouraged in grade 3), extent and resectability of primary and metastatic disease and performance status. Recent meta-analysis has demonstrated pooled sensitivity and specificity of DOTA-labeled-SSTR analogues to be 93% and 91%, respectively [52,53].

**Figure 3:**
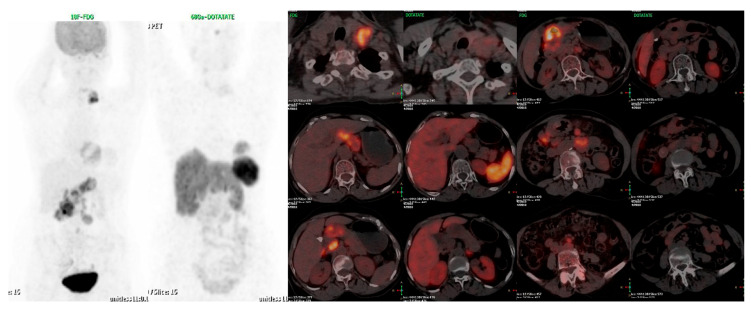
A 63-years-old female patient, recently diagnosed with Gastro-Duodenal NET, presented with pain abdomen, iron deficiency anemia and unexplained weight loss (4 kg). Gastric biopsy was suggestive of poorly differentiated NEC, WHO grade III, IHC: Synaptophysin positive; Chromogranin-A and CD-56 negative; Ki-67 index 80%. Dual tracer PET-CT with FDG and DOTATATE showed no increased DOTATATE uptake (Krenning score 0 to 1) noted in the pyloric antral mass with severe luminal narrowing and proximal dilatation with multiple enlarged lesser sac and peripancreatic lymph nodes. metabolically active gastro-duodenal primary and left supraclavicular, abdominal and retroperitoneal nodes, suggestive of minimal to no SSTR expression in the lesions. This is consistent with poor differentiation of the tumour/high Ki67 index of 80%. Hence, dual tracer PET-CT scan has potential to ascertain tumour biology and to guide best personalized management for the patient with NENs.

**Figure 4:**
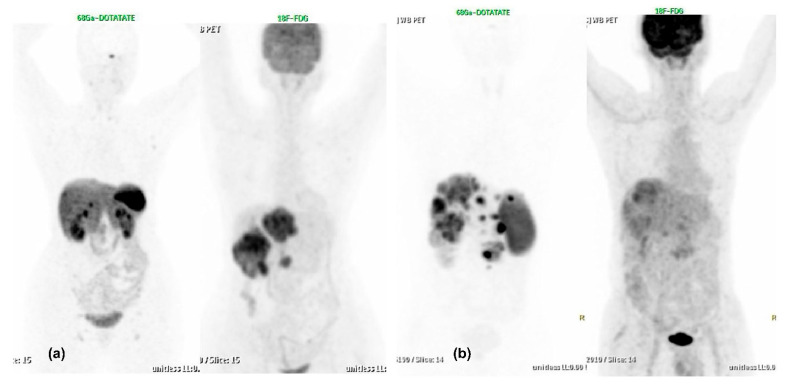
Dual tracer PET-CT scan in ascertaining personalized management for patients, selecting patients for PRRT and for prognostications. (**a**) A 55-yrs-old gentleman, case of metastatic neuroendocrine carcinoma to liver and retroperitoneal node, Ki-67 index 90% underwent dual tracer PET-CT scan which showed FDG-avid hepatic metastases involving most of the right lobe and caudate lobe; metabolically active retroperitoneal lymph nodes. The lesions show minimal to no SSTR expression –and the patient was declared unsuitable for PRRT and was referred to medical oncology. (**b**) A 42-years-old male patient diagnosed with metastatic grade II neuroendocrine tumour of duodenum, Ki-67 index 4-5%. There was disease progression after 3 cycles of cisplatin and etoposide at local place, was referred for further management and PRRT was considered. SSTR and FDG PET was done which showed intensely SSTR expressing lesions all of which showed low grade FDG activity. After 3 cycles of PRRT there was significant partial response.

A major role of SSTR based PET is selection of patients for SSTR targeted treatment—either cold or radiolabeled analogues (Figure 3 and Figure 4). Miederer and colleagues, in their study on 18 patients, found that negative IHC scores were consistent with low-grade SSTR density and hence low-grade DOTA-TOC uptake (SUVmax <10), whereas IHC scores of 2 and 3 corresponded with high grade DOTA-TOC uptake (SUVmax >15) [54]. This validates the use of SSTR PET for selecting treatment with somatostatin analogues (both non-radioactive formulations and radio-labeled analogues as in PRRT). Grade 3 NETs respond well to platinum-based chemotherapy while grade 1 and 2 NETs are refractory to chemotherapy due to low mitotic activity but responds well to SSTR targeting therapies viz. long acting cold SSAs and PRRT [55,56]. In intermediate grade NETs, there is an emerging role of Capecitabine-Temozolamide-based chemotherapy with or without PRRT [57]. As histologic grades based on biopsy from a single site may not correlate well with overall response to therapy and the fact that disease can get dedifferentiated over the course of time, it can be identified by changes in radio-tracer uptake particularly on combined FDG and SSTR PET imaging and hence can guide individualized treatment planning ([58,59]; Figure 3 and Figure 4).

**Prognostication and risk stratification:** Prognostic markers offers insight into tumor biology and natural course of the disease and deliver useful information about aggressiveness, risk of recurrence, and death, whereas predictive markers indicate benefit of a certain therapy over alternate treatment and allow physicians to tailor most effective therapeutic intervention. Hence, SSTR expression identified by SSTR-based PET examinations is both a predictive and prognostic marker as high grade SSTR expression (high SUVmax on SSTR PET) is predictive marker for successful PRRT and prognosticates a more benign disease course ([60]; Figure 4). Many studies have demonstrated SSTR positivity as an independent positive prognostic marker and negative SSTR expression as independent negative prognostic marker [61,62,63,64,65]. A recent meta-analysis including few studies comprising of 593 patients have demonstrated the prognostic importance of PET derived quantitative parameters (total tumor volume and total lesion SSTR expression) and radiomics to be more than conventional standardized uptake value [66].

### 2.5. Dual Tracer PET-CT and Its Critical Role in Neuroendocrine Tumours

The grade of NETs is an important determinant affecting effective management of neuroendocrine tumors [8]. Accurate determination of grade of NETs, hence, is of paramount importance; however, it is hampered by several potential barriers viz. common primary sites for NETs may be difficult to biopsy because of small size and difficult accessibility; intra-patient tumor heterogeneity, whereby different sites of disease in the same patient may have different grades and may respond differently to therapy; and histological grade may evolve in the same patient over time (de-differentiation) [67,68]. Imaging with PET-CT scan has great applicability in determining accreting tumor biology to guide optimum therapy (Figure 3 and Figure 4).

In “dual tracer” imaging of NET, FDG and SSTR-targeting analogues are mainly used, where FDG uptake reflects glycolytic activity and predicts aggressive tumor biology, higher grade, refractoriness to PRRT, and poorer prognosis; and SSTR targeting analogues corresponds to SSTR density, well-differentiated histology, better response to PRRT, and favorable outcome [69,70,71,72,73]. Chan and colleagues proposed a novel prognostic grading scheme incorporating dual somatostatin and FDG PET imaging—the NET-PET grade [74]. The NET–PET spectrum comprised of P1 to P5, where P1 represents SSTR positive and FDG negative are the most well-differentiated tumors, P5 represents SSTR negative and FDG positive are the most poorly differentiated tumors, while the intermediaries P2 to P4 have both SSTR and FDG positive lesions with varying degrees of differentiation. Many recent studies have identified negative prognostic value of FDG uptake in NET lesions, and in some of them, FDG uptake was the independent risk factor for shorter progression free and overall survival [50,75,76,77].

**Figure 5:**
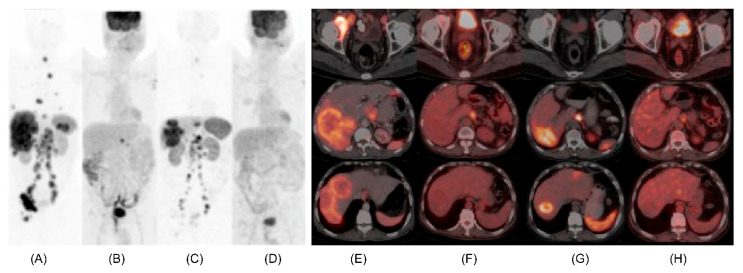
A 64-years-old lady, case of metastatic NET to liver and skeletal sites with unknown primary, presented with pain abdomen and weight loss and was referred for PRRT in view of SSTR expressing metastatic neuroendocrine tumour. Histopathology (of liver lesion) revealed metastatic NET, well differentiated, Mib-1 index 24% with synaptophysin & chromogranin positive and CDX2 negative on IHC. Baseline 68Ga-DOTATATE PET CT revealed multiple areas of increased tracer uptake (SSTR expression) in both lobes of liver and skeletal sites with no abnormal hypermetabolism evident on baseline FDG PET-CT. Follow up dual tracer PET-CT scans, after 4# of PRRT demonstrated decrease in number of smaller hepatic metastases with mild interval decrease in size of the larger hepatic lesion in the left lobe, overall partial response. Here dual tracer PET-CT scans appeared to be in agreement with the histopathological finding of well differentiated grade 3 neuroendocrine tumour (G3 NET, WHO 2017) and the findings were adequately clinically translated. (**A**,**E**): MIP and trans-axial images of baseline ^68^Ga-DOTATATE PET CT; (**B**,**F**): MIP and trans-axial images of ^68^Ga-DOTATATE PET CT after 4 cycles of PRRT; (**C**,**G**): MIP and trans-axial images of baseline FDG PET CT and (**D**,**H**): MIP and trans-axial images of FDG PET CT after 4 cycles of PRRT.

Another possible utility of dual tracer PET-CT is in discordant NETs. Adnan and Basu, in their retrospective analysis of an unselected cohort of 36 patients who were referred for PRRT, demonstrated significant correlation of dual tracer PET with overall survival while no significant correlation could be established between WHO grade and overall survival in discordant subgroup. Furthermore, dual tracer PET-CT imaging was found to be significant prognostic determinant and predictor of outcome [78]. Hence dual tracer PET with FDG and SSTR analogues in patients with discordance (between WHO grade predicted imaging findings and actual imaging findings), performed better than WHO grading, differentiation status, and IHC in prognosticating and predicting outcome (Figure 5).

### 2.6. Biodistribution and Incidental Findings on SSTR-Based PET-CT

Normal biodistribution of SSTR analogues is seen in pituitary gland, spleen, liver, adrenal glands, and urinary bladder in significant concentration, whereas thyroid, salivary, and parotid glands show faint to mild homogeneous uptake [79,80]. Lungs have high concentration of SSTR 4, and hence, generally show low grade SSTR uptake. Homogeneous uptake is seen in liver and spleen with spleen showing concentration of T-lymphocytes. Pancreatic head and uncinate process has SSTR expressing cells. In genitourinary tract, SSTRs are widely expressed in distal nephrons and collecting tubules in the kidneys and vasa recta in expresses SSTR 2 in high densities. Excretion is mainly through kidneys due to the hydrophilic nature [81,82,83].

Somatostatin receptors are widely distributed throughout the body and mediates diverse physiological functions—regulation of various hormones and neuropeptides, gastric emptying, and intestinal blood flow, which has led to increased number of incidentalomas being identified on the SSTR based PET imaging [84]. Hence, the understanding of SSTR-based PET detected incidentalomas is of crucial importance as well, in order for knowing how to deal with unexpected findings to avoid unexpected consequences. Bentestuen and colleagues, in their review comprising 2906 patients, found 133 patients to be having incidental findings that were in the thyroid gland (65), spine (30), brain (26), and breast (6) [85]. A total of 17 of 133 patients (13%) harbored malignancy on final diagnosis. Breast incidentalomas were associated with highest risk of malignancy (67%), followed by thyroid (8%) and spine (3%).

### 2.7. Future Directions and Other PET Tracers Used in Neuroendocrine Tumour Imaging

**18F-Fluoro-dihydroxyphenylalanine (FDOPA) PET:** Fluorodopa (FDOPA) is a positron emitting amino acid analogue, which is taken up into cells via neutral amino acid transporter and is an alternative PET radio-tracer in countries where [^68^Ga]-labelled SSTR PET imaging is not available. It showed superior diagnostic sensitivity to SSTR scintigraphy and is inferior to SSTR PET and comes at a higher radiation dose and cost [86,87,88]. However, FDOPA is useful in imaging SSTR-negative NET, especially medullary thyroid carcinoma (MTC) where it shows predictive value [89]. Another possible indication is nesidioblastosis in the differential diagnosis of endogenous hyperinsulinemia, pheochromocytoma, and paraganglioma [90,91].

**Glucagon like peptide-1 receptor (GLP-1R) imaging:** GLP-1R is another targetable peptide hormone receptor and is mainly located on pancreatic beta cells; the gene is an important tracer for imaging insulinomas which shows low SSTR expression and are challenging due to their small size and proximity to kidneys [87,92]. As natural GLP-1 ligand is rapidly degraded by dipeptidyl peptidase—4 (DPP4), the DPP 4 resistant GLP-1 analogue Exendin-4 was developed [93]. [^68^Ga]Ga-DOTA-Exendin-4 PET-CT showed improved diagnostic accuracy of 94% against 68% for [^111^In]In-DOTA-Exendin-4 SPECT CT and 3 Tesla MRI [94]. However, since malignant insulinoma expresses limited amount of GLP-1R, they often express SSTR and can be imaged with SSTR based analogues [95].

**Fibroblast activated protein (FAP) / FAP Inhibitors imaging:** FAP is a serine protease, overexpressed on the cell surface of activated fibroblasts, particularly cancer associated fibroblasts in tumor stroma [96]. [^68^Ga]Ga-FAPI-4 has low nano-molar affinity to FAP and is almost completely internalized, has a rapid blood clearance, and showed excellent image contrast [97]. FAPI-based imaging could be particularly interesting in imaging small intestinal NETs, which are characterized by extensive fibrosis surrounding primary tumor and mesenteric metastases [98].

**C-X-C motif chemokine receptor 4 (CXCR4) imaging:** CXCR4 is over-expressed on differentiated SSTR 2 negative NENs [99,100]. [^68^Ga]Ga-Pentixafor in direct comparison with [^68^Ga]Ga-DOTATOC and FDG PET-CT in 12 patients with GEP-NENs showed obvious diagnostic accuracy of [^68^Ga]Ga-DOTATOC (92%) followed by FDG (83%) and 50% for [^68^Ga]Ga-Pentixafor [101]. Being negative in all grade 1 patients, the diagnostic utility of [^68^Ga]Ga-Pentixafor increased with increasing tumor grade (52% in grade 2 and 80% in grade 3), but since all CXCR4 positive lesions also showed high FDG uptake there is, at present, no additional value of CXCR4 based imaging in NENs.

**Glucose dependent insulinotropic polypeptide receptor (GIPR) imaging:** GIPR is a member of gut peptide family, showing similar characteristics as GLP-1 including inactivation by DPP4 [93]. There is low grade expression of GIPR on normal cells while they are over expressed in insulinoma, gastrinoma, as well as non-functioning pancreatic, ileal, lung NENs [102]. Moreover, GIPR imaging is an attractive target for small fraction (approx. 10%) of NEN which do not express SSTR or GLP-1R with promising results on pre-clinical data.

**Cholecystokinin 2 receptor (CCK2R) imaging:** CCK2R-based imaging constitutes an attractive alternative target for peptide-based molecular imaging in MTCs (as they have low SSTR expression) [103]. [^68^Ga] labelled minigastrin analogue MG48 ([^68^Ga]Ga-PP-F11) PET-CT initially detected MTC lesions in a patient in 2016 and many CCK2R peptides are being evaluated for evaluation of MTC patients [104,105].

**Artificial intelligence tools:** Radiomics, defined as use of advanced computer analysis and deep-learning techniques to find and quantify imaging, have potential for more differentiated grading and even prediction of treatment response. In NENs, radiomics have mainly been evaluated in pancreatic NEN and were successfully used in several studies to differentiate low-grade from high-grade tumors and pancreatic carcinoma [106,107]. One study even demonstrated a correlation between developed radiomics nomogram and the proliferation markers (Ki-67 index) and mitotic count [108].

## 3. Conclusions

Somatostatin targeting PET CT is an important tool in diagnosing, staging, therapeutic decision-making, and treatment response evaluation of neuroendocrine tumors, and has demonstrated high statistical sensitivity and specificity. “Dual tracer” (SSTR and FDG) PET-CT scan has proven to be quite promising in ascertaining overall tumor biology (including inter- and intra-tumoural heterogeneity) through non-interventional approach and is pivotal to the concept of personalized and precision oncology. “Dual tracer” PET CT along with WHO grading is the most important determining factor in patient selection for PRRT, chemotherapy and “sandwich chemo-PRRT” protocol, and also finds use in prognostication and risk stratification of neuroendocrine tumors. Although many novel tracers are on the horizon for neuroendocrine tumors, the clinical utility of SSTR targeting PET CT and that of “dual tracer” imaging still largely remains unchallenged.

## Figures and Tables

**Table 1:** Clinical scenarios and appropriateness use criteria for somatostatin receptor imaging in Neuroendocrine Tumours [39].

Scenario No.	Description	Appropriateness	Score
1	Initial staging after histologic diagnosis of NETs	Appropriate	9
2	Localization of primary tumor in patients with known metastatic disease but unknown primary	Appropriate	9
3	Selection of patients for SSTR-targeted PRRT	Appropriate	9
4	Staging NETs before planned surgery	Appropriate	8
5	Evaluation of mass suggestive of NET not amenable to endoscopic or percutaneous biopsy (e.g., ileal lesion, hypervascular pancreatic mass, mesenteric mass)	Appropriate	8
6	Monitoring of NETs seen predominantly on SSTR PET	Appropriate	8
7	Evaluation of patients with biochemical evidence and symptoms of NET without evidence on CI and without prior histologic diagnosis of NET	Appropriate	7
8	Restaging at time of clinical or laboratory progression without progression on CI	Appropriate	7
9	New indeterminate lesion on CI, with unclear progression	Appropriate	7
10	Restaging of patients with NETs at initial follow-up after resection with curative intent	May be appropriate	6
11	Selection of patients with nonfunctional NETs for SSA treatment	May be appropriate	6
12	Monitoring in patients with NETs seen on both CI and SSTR PET with active disease and no clinical evidence of progression	May be appropriate	5
